# The Foreign Journals: Midwifery, and Diseases of Women and Children

**Published:** 1841-10

**Authors:** 


### MIDWIFERY, AND DISEASES OF WOMEN AND CHILDREN.

### *Secretion of Milk after Menstruation.* By Dr. Wehr, of Cassel.

M. H. a labourer's wife, 30 years old, of ordinary size, menstruated first in her eighteenth year, having previously enjoyed good health. Her catamenia continued quite regularly to the time of her marriage in her twenty-eighth year. Ten months after marriage she had a healthy child which she suckled. Since that time, now six years ago, she has never been pregnant, but after menstruation, which occurs regularly every four weeks, she has always had a burning sensation in the breasts, both of which swell up, become hard, and secrete more or less milk. Some days before the reoccurrence of menstruation, the breasts again diminish, and the secretion gradually ceases.

*Casper's Wochenschrift. Mai* 8, 1841.

---

### *On softened, encysted Tubercles in the substance of the Uterus, as a cause of difficult Labour.* By Professor Osiander, of Göttingen.

The case here recorded differs from almost all others in which labour has been impeded by the pressure of tumours. Parturition was not impeded merely by their mechanical action, nor was the pelvic cavity contracted by their presence. They produced an injurious effect by paralyzing the action of the uterus and preventing the expansion of its fibres. The obstruction to labour thus caused was quite as great as if the pelvis had been contracted, and delivery could be effected only by mutilating the child and employing the blunt hook.

The patient was a woman, forty-five years old, who had suffered from scrofula in her infancy. She was a person of unhealthy aspect, had already miscarried twice, but had never given birth to a living child. When seen by Professor Osiander, she had been twenty-four hours in labour, her strength was much exhausted, and she was very low spirited. The head of the child was felt to be very high up in the pelvis, and the membranes were still entire, though the os uteri was freely dilated.

After waiting for some hours, during part of which time the uterine action had been energetic, the head came somewhat lower down, and Professor Osiander ruptured the membranes. The head, however, remained above the brim of the pelvis, where it presented in the oblique diameter with the posterior fontanelle directed towards the right sacroiliac synchondrosis. An attempt to bring down the head with the forceps was unsuccessful, and it was next sought to deliver the patient by turning. The hand of the operator could touch the ribs, but it was found impossible to reach the feet, for a sort of stricture in the middle of the uterus rendered all attempts to carry the hand as far as the abdomen of the child, unavailing. Changing the position of the patient and placing her on her knees did not diminish the difficulties, for the contraction seemed to occupy alike all the walls of the uterus, and neither the right nor left hand could penetrate beyond it.

A second unsuccessful effort was made to deliver with the forceps; and then, after waiting for half an hour, Professor Osiander proceeded to perform craniotomy, and to extract the child by means of the blunt hook. This was not effected without great difficulty. It was necessary to introduce the hand, in order to remove the placenta, which was not adherent, but merely retained by an irregular contraction of the uterus. No serious hemorrhage followed delivery, but the uterus never contracted properly, and the abdomen continued much distended. The patient lay in a listless condition, making no complaint of pain, but with a quick pulse and tumid abdomen which were thought to indicate the propriety of venesection. No relief followed its employment, the patient was soon afterwards attacked with vomiting, and died on the third day after delivery.

On a post-mortem examination, no traces of peritonitis were found; but the whole right side of the abdomen was occupied by the enormously large uterus. The substance of that organ was nearly three fingers thick, beset with hard swellings like eggs, of a somewhat oval form, and filled with a yellow caseiform matter resembling pus, the liquid parts of which had been absorbed. These large tubercles, about nine or ten in number, were invested with a fibrous envelope. They projected on the posterior and external surface of the uterus, so as to render it uneven. Many smaller bodies of the bigness of cherries were imbedded in the uterine parenchyma, and on a section being made of them were seen to be made up of concentric fibres; thus resembling in structure the ordinary fleshy tubercles of the uterus. With the exception of partial ossification of the left ovary, the above was the only morbid appearance of moment, and to this state of the uterus the difficulty experienced in introducing the hand must be exclusively attributed.

*Hannoversche Annalen.* v Band. 15tes Heft.

---

### *On the Employment of Cold Affusion in the Treatment of Acute Hydrocephalus.* By Dr. MÜNCHMEYER, of Verden.

DR. MÜNCHMEYER observes that the medical world is greatly divided in opinion as to the value of this remedy; some persons greatly extolling its efficacy, while others regard it as altogether useless. He considers it to be a most important remedy, and one which will often save life when all other means have been useless. One great reason why cold affusion has met with so few supporters is to be found in the misconception which has prevailed with reference to the proper time for using it. It is certainly not always advisable to resort to it, and it should never be forgotten that its mode of action differs essentially from that of cold when kept constantly applied to the head. In the employment of cold affusion it is the secondary action of cold, as well as the sudden shock to the system produced by the mode of its application from which benefit is expected, while in the case of cold lotions to the head it is the primary action of cold which is obtained. Cold affusion then must not be looked upon as a directly antiphlogistic remedy, nor is its employment indicated during the early inflammatory stages of hydrocephalus, but rather when effusion, the consequence of inflammatory action has taken place, and a tendency to paralysis exists. After the subsidence of the violent symptoms of the disease, and when the patient has sunk into a comatose state, with a pale countenance occasionally suffused with a flush, dilated pupils, strabismus, and slow pulse, this remedy will frequently prove of excellent service.

In order, however, for benefit to be derived from it, it must be employed in an efficient manner. Dr. Münchmeyer directs that the patient should be taken out of bed, stripped of his clothes, and wrapped up in some simple covering (if waterproof the better), which leaves only his head exposed. He should then be placed in a sitting posture in a bath or tub, and the person who administers the affusion should mount on a chair and pour cold water upon his head, in a moderate stream from the height of five or six feet. This may be continued for a minute or two, and repeated twice or thrice. The patient should then be wrapped up in a warm sheet and placed in bed, where he should remain till it is thought proper again to have recourse to the remedy. At first, it will probably be requisite to repeat the affusion, in the course of an hour and a half or two hours; but as the patient improves the interval may be longer, so that at last it will not be necessary to employ it above two or three times daily.

The immediate effect of cold affusion is, that the patients awake from their comatose condition and begin to cry violently, which they continue to do so long as the water is poured upon them. They afterwards appear exhausted and pale, the skin is cool, the pulse small and very frequent. When placed in bed they usually fall into a dose, the pulse becomes more regular, and the warmth of the skin returns. By degrees, as with the repetition of the remedy the patients improve, they begin to have sound sleep, from which they awake in the possession of all their senses, recognize those by whom they are surrounded, and cease to squint. At the same time too a sweat, frequently of a critical nature, breaks out upon the whole body, and during its continuance the employment of cold affusion is very hazardous. The patient's sleep becomes more refreshing, and the comatose condition recurs at longer intervals; he begins to notice what goes on around him, the head regains its natural temperature, and the febrile symptoms disappear. The employment of affusion must, however, still be continued for some days, since relapses very frequently occur.

The paper is illustrated by five cases. In three the employment of cold affusion was perfectly successful, in one it produced temporary amendment, and the death of the patient was to all appearance owing to the apathy of the parents, who neglected to persevere in the treatment, while in the fifth convulsions and death followed affusion while the patient was perspiring profusely.

*Hannoversche Annalen.* Band v. Heft 4.

---

*On the Immersion of Children apparently still-born in Cold Water.*
By Dr. Scholer, Assistant Physician of the Berlin Lying-in Institution.

Nothing more need be said of this paper than it contains two well-detailed cases, and alludes to several others, in which this measure was successfully adopted, after all the ordinary means had failed of reanimating the infant. The evidence adduced is certainly sufficient to warrant the adoption of the plan as a last resource after less violent measures have been tried in vain.

*Medicinische Zeitung. April* 28, 1841.

